# South West Orthopaedic Club

**Published:** 1987-08

**Authors:** 


### South West Orthopaedic Club

### Meeting at Hereford Saturday May 10th 1986

### ORTHOPAEDIC FELLOWS OF THE ROYAL SOCIETY AND THEIR CONTRIBUTIONS TO SURGERY —

Mr A. H. C. Ratliff,
Bristol

Mr Ratliff described the founding of the Society in 1660 in the time of Charles I. Initially, entry to the Society was unlimited, but in 1847 it was restricted to Scientists who had done a particularly original work and more recently entries have been limited to 40 each year. The highest award of the Society is the Copley medal, first awarded in 1731. Mr Ratliff then went on to speak of Fellows with a particular interest in orthopaedics.

**Percival Pott** (1714–1788) was made an FRS in 1764. He is most famous for the fracture of the ankle named after him, although he himself sustained compound fracture of the lower tibia in 1756. He was a surgeon in the pre antiseptic era and amongst other things described Pott's puffy tumour and Pott's paraplegia.

**John Hunter** (1728–1793) was made an FRS in 1767. He was a surgeon at St. Georges Hospital and the founder of the Hunter museum. It was probable that he was the founder of Modern Scientific Surgery. Mr Ratliff showed two specimens from the Hunter museum.

**Astley Cooper** (1768–1841) was made an FRS in 1802. He was a lecturer in surgery at Guy's Hospital and was famous for excising a sebaceous cyst from the scalp of George IV. He was president of the Royal College of Surgeons in 1836 and was awarded the Copley medal for his work on the incision of the tympanic membrane for deafness in children. He described the manipulation of fractures and dislocation in diseases of the breast and testes. In 1822 he described the blood supply of the head and neck of the femur—few people realise this was described so early.

**Benjamin Brody** (1738–1762) was both President of the Royal College of Surgeons and President of the Royal Society. He was made an FRS on the basis of his work on the dissection of the "Foetus with no Heart". He, of course, described Brody's abscess and tumour and started the Fellowship examination. He was the first President of the General Medical Council.

**James Paget** (1814–1899) was made an FRS in 1851. He was also President of the Royal College of Surgeons. His work for his FRS was on the freezing of the albinum of the egg.

**Joseph Lister** (1827–1912) was an Edinburgh Surgeon who was made an FRS in 1860. His work on the bacterial contamination of wounds resulted in his publication in 1867 of his antiseptic technique.

Lastly, Mr Ratliff spoke of **John Charnley** (1911–1982). He was the oldest of these surgeons to be made an FRS in 1975 at the age of 64. Mr Ratliff reminded members of the many contributions made to surgery by John Charnley starting with his closed treatment of fractures continuing with his compression arthrodesis and his more famous work on arthroplasty of the hip, including both the biomechanical research and his later work on asepsis in the operating theatre.

### EARLY EXPERIENCE WITH ISOELASTIC CEMENTLESS TOTAL HIP REPLACEMENT

Mr Salman Ali
Oswestry

This paper although read by Mr Ali was on behalf of Mr Hoyle and Professor O'Connor.

Mr Ali described the early work by McKee and the later use of cement. He discussed the failures which are due to the presence of cement. He described the technique for the implantation of the isoelastic prosthesis. He noted this was a demanding technique using complicated jigs. Following the operation the patients were expected to be partial weight bearing for three months. 170 patients had had the operation in Oswestry but Mr Ali reviewed the first 100 who had been followed up for more than one year. The majority of patients had osteo-arthritis being 18% with rheumatoid arthritis and 10% miscellaneous disorders. He stated that 90% of the patients had good pain relief, the remaining 10% having some discomfort. Flexion and other movements of the hip were all good. The complications were one deep vein thrombosis and one technical error and in only one case was there femoral loosening. One patient had a revision with a long stem. A lively discussion followed this paper during which it was suggested by members that the originator Morscher, had given up the operation.

### POSTERIOR SPINAL FUSION WITH SCREW FIXATION

Mr Vic Seal
Hereford

Mr Seal described his experience with fusion of the spine using the boucher technique. He suggested that the posterior approach was less likely to give rise to long term complications than the anterior approach and the technique was simpler. He operated only on "virgin spines" and warned members of inappropriate symptoms and signs. He stressed the importance of discograms in localising the area in the level of the spine but during later discussion admitted that he felt most of the pain was arising in the posterior facet joints. He also noted that it was unnecessary to obtain bone from the iliac crest, often a source of continuing discomfort following operation, sufficient bone being obtainable from the spine locally.

He described 51 patients, 26 were single and 25 were two level fusion with a follow-up of an average of 4.2 years. Most were aged between 30 and 50 years. Excellent results were obtained in 58%, good in 27%, fair in 4% and 11 failures. He stated that 85% of those with a single fusion were significantly improved. The complications included laminal fractures in two, transient leg pain in a further one and superficial sepsis in six patients. Late screw fracture occurred in one. He had no instances of late spinal stenosis and no instances where symptoms appeared to have occurred later above the level of the fusion. A lively discussion again followed this paper and it was suggested that a pre operative assessment using facet block might be more appropriate than discograms.

### THE FLAIL ARM SPLINT

**Mr Sam Mullick**
**Bristol**

Mr Mullick stated that during the pridie Memorial Lecture in Bristol last year, Dr Wynne-Parry had reported a high usage and satisfaction amongst Stanmore patients given the Flail Arm Splint. It had been his impression that patients in the South West were less satisfied with their splints and he used them less frequently. He then went on to analyse 48 patients who had been supplied with the Flail Arm Splint. All of these patients had been carefully fitted by a Prosthetist and had had training in the use of the splint. He found that 70% of the patients did not wear their splint. The wearing of the splint had no effect on the pain felt from the shoulder but he did note that sometimes patients with a Flail Arm often held their splint as if the shoulder felt loose. He suggested that there may be in a small number of cases indication for fusing the shoulder as well as prescribing the splint. He suggested that it all amounted as to whether the patients felt they had a fundamental gain from using the splint.

### THE USE OF KIRSCHNER WIRES AS AN EXTERNAL FIXATOR IN THE TREATMENT OF HAND INJURIES

**Mr Ian Reynolds**
**Hereford**

Mr Reynolds described the use of K wires together with blobs of cement in the stabilisation of fractures of the hand. He showed that this was a very easy and inexpensive technique which can be used in the accident department with great effect.

### APPROPRIATE TECHNOLOGY IN ORTHOPAEDICS IN THE DEVELOPING WORLD

**Mr A. L. Eyre-Brook**
**Bristol**

From his wealth of experience of working abroad over many years, Mr Eyre-Brook gave a fascinating insight into the problems of orthopaedics in the developing world. He stressed the need for simple and strong aids such as wheelchairs made from local commodities which can be easily replaced. He showed how expensive and elaborate gifts of buildings are a liability diverting resources from the primary care in the community where they are most needed. He spoke of the need for training surgeons in their own country using patients with relevant diseases.

### GARY HAMPSON MEMORIAL PRIZE LECTURE — "The Mechanical Influences of Ligament Prostheses"

**Mr Paul Cooke**

Reconstruction of the anterior cruciate ligament is increasingly performed by surgeons in an attempt to restore normal function to the knee joint, whilst at the same time delaying the development of degenerative changes. Traditionally such procedures have involved transferring tissues such as fascia, tendon or meniscus, although each has disadvantages either in terms of the time taken to harvest the graft, or by increasing the instability of the joint by removing healthy tissue. For this reason, a variety of substitutes have been developed which use fine synthetic fibres as a permanent or ingrowth prosthesis. Each has been designed to match or exceed the ultimate tensile strength of the naturally occurring ligament, but few closely resemble the load deformation characteristics of the original structure, although the importance of this in the development of neoligament with normal paramenters has been shown. No product attempts to reproduce the recoverable elastic properties of ligaments.

Since such characteristics may be important in terms of both the function of the joint and the development of degenerative changes, a study was performed in which implants of differing mechanical characteristics were produced and implanted into a large animal model.

The project comprises a natural progression to the work of Gary Hampson who demonstrated the relationship between the morphology of ligaments and biomechanical behaviour. It incorporates many of the techniques which he developed, combined with advances in material sciences which now allow the development of these implants.

An initial study was undertaken in which the tensile properties of the ovine cranial cruciate ligament were defined in terms of the overall mechanics, the mechanics of the bundles of fibres, and finally of the collagen fibrils themselves. Using this data a series of prostheses were developed for implantation. By combining differing arrangements of woven fine fibres around elastomeric cores, three implants were produced which whilst made of the same materials had distinct mechanical characteristics. Thus one was stiffer than normal one was matched to the characteristics of the naturally occurring ligament, and one was laxer than normal.

Next a group of animals were trained to walk in a controlled fashion over a force plate in order to allow subsequent functional assessment to be undertaken. The animals were randomly allocated to four groups (each of six sheep) which underwent surgery as follows.
Group A — excision of the cranial cruciate ligament — no substitution.
Group B — excision of the cranial cruciate ligament — stiff prosthesis.
Group C — excision of the cranial cruciate ligament — matched prosthesis.
Group D — excision of the cranial cruciate ligament — lax prosthesis.

The animals were encouraged to mobilise freely after surgery and at intervals of two months were walked across the force plate to determine the degree of lameness which persisted.

After six months it could be seen that the animals which had a stiff or matched implant (groups B & C) bore significantly ($p<0.01$) more weight through their hindlimbs than the animals with no prosthesis or a lax prosthesis (groups A & D). However, when the articular surfaces were examined for signs of cartilate fibrillation and joint degeneration, there was evidence of significantly more damage ($p<0.05$) in the group with a stiff prosthesis (gp B) than the matched group (gp C). This suggests that although there was no difference in terms of early function between the stiff and matched implants, the stiff implants appeared to predispose to joint damage. This may result from producing excessive compressive forces between the joint surfaces. The matched implants conveyed relative protection from degenerative changes.

Since all prosthetic ligaments are stiff on cyclical loading, this has important implications. Firstly we should be aware that there is a possibility that such implants, and indeed biological transfers have tential to induce rather than prevent degeneration. Secondly that the development of a biomechanically matched implant, although fraught with many difficulties in the complex environment of the human knee, appears to be a worthwhile objective. Work aimed at development of such a prosthesis is currently being undertaken by a group of engineers, orthopaedic surgeons and veterinary surgeons from Bristol and Oxford.
done by the consultant but by a member of his team. Patients referral letters were dated between 0 and 141 days after operation with 74 within 20 days of amputation. They first attended the Centre between 10 and 166 days after amputation, 55 within 30 days and 70 within 40.

Criteria for assessing the quality of an amputation stump include whether the wound is healed, whether the stump is conical or bulbous, whether it is optimum length, when it is felt suitable for fabrication of temporary and permanent prostheses, and in the cases of below knee amputation, whether an ischial bearing AK/BK pylon is necessary initially and at what stage it can be cast for a PTB pylon.

The single CHOPART amputation (for congenital deformity) was excellent on presentation, as was one of 2 Symes amputations but the other was not fully healed. There were 52 below knee amputation stumps in this series and it is stressed that patients with an amputation at this level are very much less disabled than those with more proximal amputation. Only 22 (42%) were considered an ideal length (5–5½") at presentation. Twenty-two (42%) were not fully healed. In some cases it appeared that healing had been delayed or skin grafting had been performed in order to preserve an unnecessarily long stump. Twenty-one (40%) were an unsatisfactory shape. In some cases this was due to terminal oedema for which faulty bandaging sometimes appeared responsible but in others it was due to inadequate tapering of the posterior muscle flap in which the residual limb may remain a bad shape for years. Overall 41 below knee stumps (69%) were in some way unsatisfactory but if minor variation in length were accepted the number of entirely satisfactory stumps rose to 15 (29%) and 29 (55%) were considered suitable for PTB pylons when first seen.

One patient had bilateral through knee disarticulations and there were 15 Gritti Stokes amputations in this series. Three of these were not healed on presentation. Amputation at this level produces a strong residual limb but the prostheses available are ugly and unacceptable to many patients.

Twenty-eight amputations had been performed above the knee of which 9 were less than 4 or more than 6" from the joint. Five were unhealed on presentation and another 5 were swollen distally, overall 15 stumps (54%) were unsatisfactory in some way but only 3 (11%) were unsuitable for immediate measurement for a pylon.

Over the whole series of 100 amputation stumps, 30 were good, 56 were bad and 14 were ugly when first seen at the Centre. These findings are similar to those of other surveys in different parts of this country and show no improvement since the report by the British Orthopaedic Association in 1972 quoted recently by Professor McColl.

